# Medicaid Enrollment following the ACA Eligibility Expansion amongPersons using Homelessness Services: An interrupted time-series analysis

**DOI:** 10.21203/rs.3.rs-6247440/v1

**Published:** 2025-09-01

**Authors:** John S. Palatucci, Sujoy Chakravarty, Evan S. Cole, Jose Nova, Taiisa Kelly, Julie M. Donohue, A. Everette James, Joel C. Cantor

**Affiliations:** Rutgers University; Rutgers University; University of Pittsburgh School of Public Health; Rutgers University; Monarch Housing Associates; University of Pittsburgh School of Public Health; University of Pittsburgh School of Public Health; Rutgers University

**Keywords:** Unhoused Persons, Medicaid, Patient Protection and Affordable Care Act, Housing Instability

## Abstract

**Background:**

Persons experiencing homelessness bear high rates of morbidity, injury, and mortality. Medicaid offers an opportunity to provide support, but barriers persist to enrolling and maintaining enrollment among this vulnerable population. The objective of this study was to determine the initial and longer-term effects of the Affordable Care Act (ACA) Medicaid eligibility expansion on Medicaid enrollment among persons observed to be unhoused or housing insecure through recorded housing services utilization.

**Methods:**

We applied interrupted time-series analyses of linked administrative data from two expansion states – New Jersey (NJ) and Pennsylvania (PA); Homeless Management Information System data were linked to monthly Medicaid enrollment files of non-elderly adults (aged 18–64) utilizing shelter, street outreach and other housing assistance from January 2011 to December 2016. The study outcome was a binary measure of Medicaid enrollment status in month of Homelessness Management Information System service exit overall and stratified by race/ethnicity.

**Results:**

ACA Medicaid eligibility expansion was associated with a level change in the likelihood of enrollment of 7.5 percentage points (pp) in NJ and 8.5 pp in PA. The trend in enrollment post-expansion also increased by 0.6 pp/month in NJ. Compared to no homeless-related service use in the year, being recorded with one month with a shelter stay or other homelessness assistance services in the year was associated with a higher likelihood of Medicaid enrollment (14.8 pp higher in NJ and 6.9 pp higher in PA), and likelihood of enrollment was highest when two or more months with homelessness assistance services were used in the year (18.6 pp higher in NJ and 12.8 pp higher in PA). However, the effect of the policy change attenuated back to the pre-ACA trend in both states by the end of 2016. Results were similar across race/ethnicity stratifications.

**Conclusions:**

We found significant increases in the likelihood of Medicaid enrollment after the ACA Medicaid expansions in the months immediately following the expansion. Additional months with homeless shelter stay or other housing services for unhoused persons were associated with a higher likelihood of Medicaid enrollment; this suggests the need for further investigation into the potential of leveraging staff-client relationships at homelessness assistance programs during future health policy initiatives.

## INTRODUCTION

On a single night in 2024, there were more than 770 000 persons experiencing homelessness in the United States.^1^ Hundreds of thousands more cycle through episodes of homelessness each year with the US Department of Housing and Urban Development most recently counting 1.4 million persons as being homeless based on service utilization (including emergency shelters and related programs) in 2022.^2^ Those experiencing homelessness face increased rates of mortality and infectious disease,^3,4,5^ often exhibit poorly managed chronic conditions,^6,7^ and bear heightened risk of criminal victimization and other trauma exposures.^8,9^ Recent policy proposals have included recommendations to use Medicaid mechanisms to support health and well-being among this population with complex needs.^10^ These have included providing tenancy supports, designed to maintain existing housing placements,^11^ and facilitating greater access to permanent supportive housing, which provides housing and supportive services to households in which at least one member has a disability.^12,13^

In 2023, twenty-eight states were administering Medicaid-funded housing-related support for persons experiencing or at risk of homelessness.^14^ The effectiveness of these initiatives relied on the assumption that persons experiencing homelessness had stable Medicaid enrollment. Medicaid eligibility has typically included categorical eligibility requirements for adults without dependents. However, many persons experiencing homelessness or housing insecurity face barriers to enrollment, such as distrust or disappointment with public systems, limited English language literacy (especially among non-native speakers), difficulty maintaining documents required for enrollment, redetermination and documentation of disability, and/or lack of stable contact information.^15^ These barriers to enrollment were recognized in the lead-up to the Affordable Care Act (ACA) Medicaid eligibility expansions when Medicaid income eligibility thresholds were increased to 138% of the Federal Poverty Level, and categorical eligibility limitations eased.^16^ Adults experiencing homelessness were expected to become eligible for Medicaid based on their income alone.^17^ Regarding the known barriers to enrollment, there were calls, at the time, for targeted outreach to enrollment, enhanced access to key service provisions of health services for persons experiencing homelessness, and for addressing silos between housing and healthcare systems.^17,18^ However, these efforts have been largely untested; prior studies of the ACA Medicaid eligibility expansion on enrollment among persons experiencing homelessness have been limited to unadjusted measures of changes in rates of Medicaid enrollment observed when persons experiencing homelessness seek medical treatment or were conducted after a limited post-expansion observation period.^19^

This study aimed to determine the extent to which Medicaid eligibility expansions increased enrollment of persons who use homelessness assistance and other housing services and to build upon prior studies by evaluating adjusted enrollment trends over an extended follow-up period. Based on evidence from an interrupted time series analysis of the effect of the pre-ACA Medicaid expansion in Massachusetts, which expanded eligibility in 1997,^20^ we hypothesized that persons who use homelessness assistance and other housing services would have a greater likelihood of Medicaid enrollment following the ACA expansion, compared to their pre-ACA levels. According to CMS, overall Medicaid and CHIP enrollment increased by 39.8% in NJ and 22.3% in PA by December 2016 over pre-ACA levels. However, given the barriers mentioned above to stable Medicaid enrollment (such as distrust of public systems), we also expected that Medicaid enrollment would be lower among those in our sample using homelessness assistance services, such as sheltered or unsheltered homelessness assistance services, as observed in HMIS service utilization (see methods for additional details) and that there would be disparities in changes in Medicaid enrollment by race/ethnicity.

## Methods

### Data

This project relied on a data linkage of the Homelessness Management Information System (HMIS) and Medicaid Management Information System (MMIS) from New Jersey (NJ) and Pennsylvania (PA). This included data from 19 of 21 counties in NJ and 44 of 67 in PA.[1] The HMIS is the US Department of Housing and Urban Development (HUD) system “to collect client-level data and data on the provision of housing and services to individuals and families experiencing or at risk of homelessness.”^21^ The HMIS records federally funded housing, homelessness, and related supportive services provided to persons experiencing or at risk of homelessness (and may also capture ancillary services provided through non-HUD funding sources). HUD requires local homelessness assistance providers and service coordinators that distribute HUD funds to use the HMIS. State Medicaid agencies and the Centers for Medicare & Medicaid Services rely on MMIS to track enrollment, utilization, spending, and demographic information of enrolled Medicaid beneficiaries. Under the auspices of the respective housing and Medicaid state agencies in NJ and PA, novel data linkages of HMIS data and monthly MMIS enrollment records were performed in each state.

The person-month analyses in this article use the linked NJ and PA data for 2011 to 2016 for non-elderly adults (aged 18 to 64) who used at least one HMIS-recorded service during the study period (see table, SUPPLEMENTAL TABLE 1, for definitions of the service types recorded within the HMIS data).^22^ We further restricted the sample to those who did not utilize Permanent Supportive Housing (PSH) services because it is likely that individuals in PSH, many of whom qualify for PSH based on disability status, may differ systematically from all other clients with recorded services in HMIS. However, the Permanent Supportive Housing population was included in sensitivity analyses reported in SUPPLEMENTAL TABLE 2.

#### Study Outcome

Medicaid enrollment status, including partial benefit and regardless of Medicaid eligibility category, was evaluated during a client’s observed HMIS service exit, e.g. the month in which a person left a shelter stay. The study outcome was coded as a binary measure (i.e., “1” if the individual was enrolled in Medicaid in the month of service exit; “0” otherwise); service exit was chosen to capture any impact from participating in any housing services immediately preceding Medicaid enrollment, and HMIS demographic data was relatively more complete at exit compared to data collected at admission. Given that a subset of persons who used homeless and housing services may have been previously eligible for Medicaid (e.g., through the “Aged, Blind or Disabled” eligibility category) but not enrolled before expansion, this approach captures gains in enrollment through the ACA expansion’s eligibility expansion on the newly eligible as well as the possible effect of outreach, as called for in policy and scholarly literature at the time,^17,18^ to individuals who may have previously been eligible (i.e., the “welcome mat” effect).^23^

#### Homelessness Assistance Services

Each HMIS record includes information about the types of services the client utilizes. We consulted with housing and policy experts to identify specific “homelessness assistance services” by categorizing HMIS service use in each year.^24^ Ultimately, six HMIS recorded services were designated as homelessness assistance services: Coordinated Entry, Emergency Shelter, Day Shelter, Safe Haven, Street Outreach, and Transitional Housing (although coordinated entry was not available in earlier years and was subsequently excluded from the analyses). Analysts classified clients in our datasets as having zero, one, or two or more months with evidence of homelessness assistance services observed in the year. In regression models, those with zero homelessness assistance services in each year were the reference group (i.e., those using other housing services not indicative of homelessness).

#### Additional Covariates

Regression models also included HMIS recorded age, gender, and HMIS recorded race/ethnicity. Racial and ethnic groupings included White-alone non-Hispanic, Black/African American-alone non-Hispanic, Hispanic, and, due to small sample sizes, a collapsed field that included non-Hispanic clients with Asian, Pacific Islander, American Indian, or multiple races recorded on HMIS.

### Statistical Analyses

Deidentified datasets were made available to two distinct analytic groups, one at Rutgers University and one at the University of Pittsburgh that separately implemented a shared analysis plan using their respective states’ datasets. Descriptive analyses in NJ and PA samples included a measurement of the unadjusted monthly trend in Medicaid enrollment among HMIS clients. We then performed interrupted time-series analyses using a segmented regression approach with a linear probability model to examine the period from 2011–2016. The NJ study included a 36-month pre-policy baseline period and a 36-month post-expansion period, and the PA study included a 48-month pre-policy baseline period and a 24-month post-expansion period.^25^

The goal of any interrupted time series (ITS) analyses is to estimate the effect of the ACA Medicaid eligibility expansion by adjusting for the trend in the likelihood of enrollment that would have occurred in the absence of the eligibility expansion (i.e., the counterfactual).^25^ Results of ITS yield both a measurement of the level change in the study outcome (i.e., a “change in intercept” of the regression line at policy implementation) and a change in the outcome trend (i.e., the “change in slope” of the regression line since policy implementation). The statistical models accounted for different policy start dates in NJ (January 2014) and PA (January 2015) by modifying variables to capture the policy change timing for each state.

The primary linear probability model, developed after visual inspection of unadjusted trends, specified as follows, was implemented to estimate changes in the likelihood of Medicaid enrollment:

(1) $$E\left(Y_{it}|(\text{post\_expansion}{)}_{t}\ldots X_{it}\right)=Pr\left(Y_{it}=1|{\left({\text{post}}_{\text{expansion}}\right)}_{t}\ldots X_{it}\right)=\beta_{1}(\text{post\_expansion}{)}_{t}+\beta_{2}(\text{month\_indicator}{)}_{t}+\beta_{3}(\text{month\_indicator}\times \text{post\_expansion}{)}_{t}+\beta_{4}(\text{homelessness\_indicative\_services\_}1\text{\_month}{)}_{t}+\beta_{5}(\text{homelessness\_indicative\_services\_}2\text{\_or\_more\_months}{)}_{t}+\beta_{0}(\text{since\_jan}2016{)}_{t}+\beta_{7}(\text{months\_since\_Jan}2016{)}_{t}+\lambda X_{tt}+\beta_{0}+\varepsilon_{tt}.$$

*Y*_*lt*_ is a binary outcome variable of Medicaid enrollment for client *I* at time *t*. Coefficients, *β*_*1*_*, β*_*2*_, and *β*_*3*_, capture the effect of the interrupted time series variables: level change post expansion change (i.e., change in intercept), the month indicator (i.e., counterfactual), and change in direction of the trend in the months since expansion (i.e., change in slope), respectively, on likelihood of Medicaid enrollment. *β*_4_ and *β*_*5*_ capture the effect of homelessness assistance services for “one month” and “two or more months” with such services in the year, respectively. *β*_6_ and *β*_*7*_ reflect the attenuation of effect observed in the year 2016, both the level change and change in trend. *λ* represents coefficients on additional covariates, *X* (i.e., demographic variables), and *ε* is the error term. The linear probability model used to implement the ITS analyses included county-level cluster-robust standard errors and county-fixed effects in each of the respective state analyses. Models were also stratified by racial/ethnic group and sensitivity analyses were performed that included HMIS records from Permanent Housing project types.

[1] New Jersey data excludes Middlesex and Bergen counties; Pennsylvania data excludes Beaver, Bedford, Berks, Cameron, Chester, Clarion, Columbia, Dauphin, Delaware, Erie, Forest, Fulton, Huntingdon, Juniata, Lackawanna, Lancaster, Philadelphia, Snyder, Sullivan, Susquehanna, Union, Wyoming, and York counties

## Results

### Descriptive Summary

Table 1 summarizes the characteristics of HMIS recorded service among non-elderly adults who were in the linked HMIS-MMIS data set by expansion period in NJ (N=294 636 person-months) and PA (N=52 278 person-months) over the 72-month study period. In both states, there was a substantial increase from the pre- to post-expansion periods — expansion began in January of 2014 in NJ and January of 2015 in PA — in the unadjusted rate of enrollment, from 49.2% to 61.0% in NJ – an increase of 24.0% – and from 52.7% to 74.1% in PA– an increase of 40.6%.

We also observed that the percentage of persons with an indication of two or more months with homelessness assistance services use was larger in the post-expansion period in NJ (18.3% to 24.6%) and a slight lower in PA (18.2% to 16.3%). Mean age did not change in NJ or PA after expansion. There was minimal change in race/ethnicity groups and gender categories between the pre- and post-expansion periods in both states.

**Figures 1:**
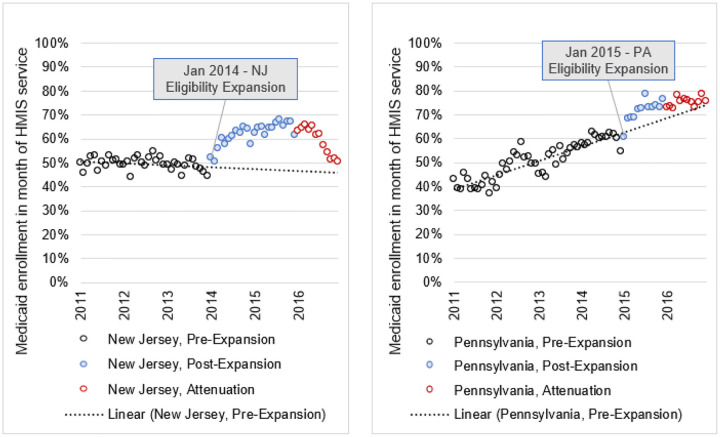
Legend not included with this version.

Fig. 1 presents the unadjusted trend in percentage of Medicaid enrollment, among non-elderly adults (aged 18–64) utilizing housing and/or homelessness assistance services, over the 72 months in the study period, 2011–2016 for each state. Prior to the ACA eligibility expansion in NJ, the percentage of Medicaid enrollment trended downward. Conversely, there was an increasing trend in the percentage of Medicaid enrollment in PA. In both states, there were distinct increases in percentage of enrollment following the ACA Medicaid expansion. After initial increases, enrollment rates in both states began to level off in 2016 and then, in NJ, rates of Medicaid enrollment declined sharply. By the end of 2016, rates of enrollment in both states returned to the trend line established during the pre-ACA period.

Percent of Medicaid enrollment among non-elderly adults (aged 18–64) using homeless or other housing services in New Jersey and Pennsylvania in month of Homelessness Management Information System (HMIS) end date, 2011–2016. Source: Authors’ analysis of linked HMIS and MMIS data in NJ and PA (see text). Abbreviations: NJ, New Jersey; PA, Pennsylvania; HMIS, Homeless Management Information System; MMIS, Medicaid Management Information System; NH, Non-Hispanic. Notes: The denominator includes individuals with observed service use in the Homeless Management Information System, who were non-elderly adults aged 18–64 years. Medicaid enrollment percentage indicated by a circle for each month of the study and shaded following ACA Medicaid eligibility expansion implementation. The callout indicates eligibility expansion (i.e., January 2014 in NJ; January 2015 in PA).

### Interrupted Time Series Analyses

Table 2 summarizes the main interrupted time series analyses where the coefficients indicate that changes in the likelihood of HMIS records linking to Medicaid enrollment records was positive in both states. The main independent variables are the level change (i.e., the shift in likelihood after the ACA expansion) of 7.5 percentage points (pp) in NJ and 8.5 pp in PA, both at *P*<.001. The change in monthly trend (i.e., change in slope increased by 0.6 pp per month in NJ (*P*<.001) and 0.2 pp per month in PA (*P*=.138). Noting the unequal follow-up duration period in both states, estimates of the combined, adjusted effect of the ACA Medicaid eligibility expansion is limited to the *12 months following expansion* was a 14.7 pp increase in NJ and a 10.9 pp increase in PA. Last, the trend absent the expansion, in NJ was −0.1 pp per month (P=.053) and 0.4 pp per month (P=.015) in PA.

Utilization of homelessness assistance services was associated with greater likelihood of being enrolled in Medicaid in both states. Compared to clients in HMIS who utilized other services (e.g., homeless prevention services), persons with one month in the year with evidence of homelessness assistance services were 14.8 pp more likely to be enrolled in Medicaid at HMIS exit in NJ and 6.9 pp more likely in PA (*P*<.001 in both states) (For a complete list of non-homeless services, see Appendix Table 1 for available HUD definitions). Among those with two or more months in the year with homelessness assistance services these figures were even larger, 18.6 pp in NJ and 12.8 pp in PA (*P*<.001 in both states). Those reporting female gender had a higher likelihood of Medicaid enrollment (12.3 pp greater in NJ and 18.6 pp greater in PA; *P*<.001 for both) than those identifying as male, transgender or non-binary. Consistent with Fig. 1, interrupted time series analysis suggests a significant change in the regression line starting in 2016 in both states (1.9 pp per month decrease in NJ and 0.4 pp per month decrease in PA; *P*<.001 for both). This change in slope during 2016 amounted to a 22.8 pp reduction in NJ and a 4.8 pp reduction in PA over 12 months, eliminating most gains from expansion in both states among persons with recorded HMIS service.

Table 3 summarizes analyses of the association of the expansion with Medicaid enrollment stratified by race/ethnicity groups. While the direction of association and level of significance of point estimates are similar across race/ethnicity categories, the increase in level-change in Medicaid enrollment was greatest among white, non-Hispanic individuals in both states, and the change in trend was greatest among those in the white, non-Hispanic group in New Jersey. Having a record of two or more months with homelessness assistance services in a year was associated with large increases in the likelihood of Medicaid enrollment across all race/ethnicity groups, except for Hispanic clients in PA — which had a limited sample available. Female gender was also associated with a higher likelihood of Medicaid enrollment across all race/ethnicity groups. Sensitivity analyses that included records from those who received permanent housing services showed similar results to the main analyses where those services were excluded (see SUPPLEMENTAL TABLE 2).

## DISCUSSSION

Our findings demonstrate that enrollment in Medicaid among adults receiving services to prevent or ameliorate homelessness increased in NJ and PA following the ACA Medicaid eligibility expansion. Using any homelessness assistance services was associated with a greater likelihood of Medicaid enrollment among HMIS clients in both the pre- and post-expansion periods (i.e., using “homelessness assistance services” as described in the Methods section). This finding is counter to our hypothesis that well-documented barriers to enrolling people experiencing homelessness would lead to a lower likelihood of Medicaid enrollment compared to persons with HMIS-recorded service in non-homelessness assistance service types. One explanation is that barriers addressed through efforts by service workers to complete Medicaid applications and outreach to individuals using shelters and similar services were more pronounced among those using homelessness assistance services.

In interpreting our results, we were concerned that dynamic changes in housing inventory or the underlying unhoused population might be driving the differences in Medicaid enrollment rates that we observed. In response, we adjusted for changes in time-invariant heterogeneity in our sample by applying geographic fixed effects.

To further check for the robustness of our results, we examined HUD’s Housing Inventory Counts. We did not observe large differences in the expansion or contraction of housing/shelter programs in either state over time, nor did we find that it is likely that housing inventory in participating programs drives the differences observed in Medicaid enrollment rates. See Supplemental Fig. 1 for supplemental analysis of HUD Housing Inventory Counts on Total Year-Round Beds for Emergency Shelter, Transitional Housing, and Safe Haven.^26^

We also examined HUD’s Point-In-Time estimates. We were concerned that differences in observed rates in Medicaid enrollment could be driven by differences in the underlying population represented in our data of HMIS records. We found that HUD’s measure of overall homelessness fell by 37.1% in NJ but increased by 1.6% in PA during our study period (2011–2016).^26^ Despite these differences, the adjusted effects of reform presented above, and subsequent losses in enrollment in 2016, were generally similar between the two states suggesting that our findings are robust to these differences in rates of homelessness between states. See Supplemental Table 3 for additional detail.

HUD-funded housing services programs, such as those observed in the HMIS data analyzed in this study, often have staff tasked with supporting health and housing services enrollment. During implementation of the initial ACA eligibility expansions in 2014 and 2015, the United States Interagency Council, a group consisting of secretaries of Housing and Urban Development, Health and Human Services, Department of Commerce, among others, developed strategy documents for enrolling persons experiencing homelessness and in using public assistance to support persons enrolling in services.^27,28^

While speculative, developing and supporting client-staff relationships in homelessness assistance programs in the implementation of policies may contribute to the observed Medicaid enrollment among this population. Administrative data does not support analyses of the mechanism through which the quality of services used may lead to higher Medicaid enrollment. Thus, further study should examine the practices of homelessness assistance providers and attitudes of people experiencing homelessness in the delivery of medical benefits and housing supports through Medicaid.

Although Black/African American adults are at especially elevated risk of experiencing homelessness,^29^ our analyses did not reveal large differences in the effect of the ACA Medicaid expansion among adults using HMIS recorded services by race/ethnicity group in either state. Differences between Black/African American, Hispanic, and other race/ethnicity groups compared to White, non-Hispanic adults in our main models are small and generally not statistically significant. While readers may find it encouraging that we did not identify racial/ethnic disparities in Medicaid enrollment following the ACA in our data, future research should evaluate if such findings are consistent in other states and examine remaining drivers of racial/ethnic disparities among persons experiencing homelessness.

We note the decline in Medicaid enrollment trend among people using homeless services starting in 2016 in both states, but more severe in NJ. Ultimately, gains made following the ACA eligibility expansion returned to the trend line by the end of 2016. According to the US Census Bureau, the proportion of Americans that were uninsured increased by 1.16% from 2016–2017, suggesting a loss of coverage in 2016. Concurrently, the proportion of Americans covered by Medicaid fell by 1.4%.^30^ This difference was more pronounced in expansion states where the proportion of respondents that were uninsured increased by 3.5%.

One possible explanation for the observed attenuation in our analysis, and the less pronounced changes in the general population reported by the Census Bureau, is that income increases associated with a strengthening economy during this period may have contributed to Medicaid coverage loss among those whose incomes rose above 138% of the Federal Poverty Level. If true, this would mean that Medicaid losses among those experiencing or at-risk of homelessness were in a precarious position where they earned too much to qualify for Medicaid, but not enough to address their housing needs without additional support.

Another plausible explanation relates to the messaging during the 2016 federal election cycle; the ACA “repeal and replace” narrative gained popularity nationwide and may have contributed to a lowering of confidence in enrollment efforts. For instance, staff members working on Medicaid enrollment outreach and HMIS clients considering the effort needed to enroll may have focused on other priorities if they suspected that coverage loss with a new presidential administration transition was imminent. Future research should examine the experiences of front-line services staff as they interact with persons experiencing or at-risk of homelessness, especially in efforts to provide health services and policy initiatives during periods when political messaging may conflict with dissemination and implementation goals.^31^

### Limitations

This work is built on novel data linkages performed in two expansion states. The advantage of this approach is that it provides valid and reliable measures of Medicaid enrollment among persons using homelessness assistance services. However, while we note significant changes after the ACA eligibility expansion, large partial effect sizes, and, to a great extent, consistency across states and race/ethnicity groups, we cannot establish a definitive causal relationship given the absence of a comparison group (e.g., non-expansion states) as would be included within a difference-in-differences framework, if such data were available.

The HMIS collects data on the use of a range of services to prevent or ameliorate homelessness and does not record experiences of homelessness *per se*. For instance, the HMIS does not record homelessness among people who do not use formal services (e.g., people who may stay with friends or family without HUD-funded programs or who purposefully avoid contact with outreach workers or services). There may also be heterogeneity in the experiences of people using HMIS-recorded services (e.g., day shelter compared to emergency shelter users), which we could not assess due to sample size limitations. Future research should investigate the varied challenges of persons experiencing homelessness, such as those who do not use HMIS-recorded services, for ways to further enhance Medicaid enrollment strategies.

We had limited ability to disaggregate race/ethnicity groups to consider the effects of those who may be at elevated risk of homelessness but who are not present in large numbers in the NJ and PA samples (e.g., Native American/American Indian). The experiences of race/ethnic groups underrepresented in our data are critical to future research regarding persons experiencing or at risk of homelessness.

We were concerned that persons using HMIS services indicative of homelessness (such as emergency shelters) could have been experiencing housing instability for months before or after they entered a shelter. Additionally, observed homelessness service utilization had a severely skewed distribution in our data. To address these concerns, we collapsed counts of homelessness service utilization annually and categorized them as described above. In doing so, the recorded homelessness services may have occurred up to eleven months before or after Medicaid enrollment, a form of contamination. Additionally, given that this is a point-in-time measure, temporary lapses in coverage provides may lead to lower estimate of enrollment when measured monthly, as opposed higher levels of aggregation, such as any enrollment in the year. Taken together, our approach provides a conservative, or lowerbound, measure of Medicaid enrollment that is sensitive to month-to-month changes.

Our study included 63 of 88 counties in two northeastern expansion US states with mid-size urban areas (e.g., Pittsburgh, PA and Newark, NJ), suburban counties, and rural areas, but does not include large urban centers (data were not available for Philadelphia, PA). While we adjusted for geographic variation with county fixed effects within each state, the nature of homelessness and efforts to enroll individuals under the ACA Medicaid expansion may not be generalizable to areas where homelessness is concentrated in large cities (e.g., Los Angeles, CA and New York, NY). The generalizability of our findings also presumes a state’s expansion of Medicaid eligibility. Further, while diverse as measured by observed covariates, our sample was limited to records with valid demographic data; this especially affected sample size in Pennsylvania.

Lastly, while unlikely to affect overall trends in measures of Medicaid enrollment, we were unable to examine existing insurance, income, and other Medicaid eligibility criteria. Future research should consider such components of enrollment to further inform differentiated enrollment strategies across HMIS recorded service categories.

## Conclusions

Our findings reflect the success of thousands of persons using homeless or other housing services who were able to enroll or maintain Medicaid enrollment following the Medicaid ACA eligibility expansions. We also found few differences in changes in enrollment rates across race/ethnicity groups. Additionally, greater contact with homelessness services was associated with a higher likelihood of Medicaid enrollment across race/ethnicity groups. The high association of contact with emergency shelters and other homelessness assistance services suggests the need to understand the importance of staff-client encounters at housing and homelessness service programs and how these encounters may drive Medicaid enrollment and sustain enrollment of persons using homeless or other housing services over time.

These findings highlight the value of investing in collaboration among persons experiencing or at risk of homelessness, state Medicaid agencies, state housing agencies, and housing providers. Future studies of these cross-sector partnerships should consider the association of Medicaid enrollment and homelessness assistance services when evaluating the effect of Medicaid eligibility redetermination procedures that were paused during the COVID-19 public health emergency on persons experiencing or at risk of homelessness.^32^ As housing transition and support services become common elements of state Medicaid demonstration waivers,^13^ it will be crucial to continue to study how the dynamic relationships between persons experiencing homelessness and homelessness assistance providers inform and affect implementation efforts.

## Supplementary Material

This is a list of supplementary files associated with this preprint. Click to download.

- ACAHomelessnessMedicaidEnrollmentSupplementalTablesandFigures2025.6.18.docx

## Figures and Tables

**TABLE 1:** Descriptive summary of non-elderly adults (aged 18–64) with Homeless Management Information System recorded service in New Jersey and Pennsylvania, 2011–2016

Study Sample	NJ		PA	
	*(n = 294 636 person-months)*		*(n= 52 278 person-months)*	
ACA Medicaid Expansion Time Period	Pre-Expansion (36 months, 2011–2013)	Post-Expansion (36 months, 2014–2016)	Pre- Expansion (48 months, 2011–2014)	Post-Expansion (24 months, 2015–2016)
Average unique persons per month	3,695.3	4,489.1	660.4	857.5
Observed as enrolled in Medicaid	49.2%	61.0%	52.7%	74.1%
Homelessness assistance services per year				
*No months with homelessness assistance service encounters*	56.4%	50.0%	33.1%	33.9%
*1 month with homelessness assistance services*	25.3%	25.4%	48.7%	49.8%
*2+ months with homelessness assistance services*	18.3%	24.6%	18.2%	16.3%
Age	40.8	40.4	38.6	38.6
Race/Ethnicity				
*Asian and other racial/ethnic groups, NH*	3.8%	2.8%	2.5%	2.7%
*Black/African American alone, NH*	49.9%	50.0%	34.1%	37.8%
*Hispanic*	18.3%	18.5%	6.6%	6.4%
*White alone, NH*	28.0%	28.7%	56.9%	53.1%
Gender				
*Female*	49.6%	47.6%	44.9%	47.5%
*Male*	50.4%	52.4%	55.1%	52.5%
*Transgender, non-binary*	<0.01%	<0.01%	<0.1%	<0.1%

Source: Authors’ analysis of linked HMIS and MMIS data in NJ and PA (see text). Abbreviations: NJ, New Jersey; PA, Pennsylvania; HMIS, Homeless Management Information System; MMIS, Medicaid Management Information System; NH, Non-Hispanic. Notes: All χ2 tests of differences in proportions significant at P<0.001. Two-sample t-test of means with equal variances statistically significant at P<0.001 in NJ but represents a difference in age of 5 months.

**TABLE 2:** Interrupted time series analyses of the ACA Medicaid eligibility expansion on Medicaid enrollment among persons experiencing or at-risk of homelessness in NJ and PA, 2011–2016

	NJ		PA	
Model (Likelihood of Medicaid Enrollment)	Coefficient (95% CI)	*P*	Coefficient (95% CI)	*P*
[β_1_] Level change post-expansion	0.075	***	0.085	***
	(0.045–0.106)		(0.055–0.115)	
[β_3_] Change in monthly trend post-expansion (per month)	0.006	***	0.002	.138
	(0.004–0.008)		(−0.001 to 0.004)	
[β_2_] Monthly trend (per month)	−0.001	.053	0.004	.015
	(−0.002 to 0)		(0.001–0.008)	
Homelessness assistance services per year				
[β_4_] *1 month with homelessness assistance services*	0.146	***	0.069	***
	(0.096–0.200)		(0.45–0.093)	
[β_5_] *2+ months with homelessness assistance services*	0.185	***	0.128	***
	(0.128–0.244)		(0.110–0.145)	
Race/ethnicity (white NH reference)				
_1_] *Asian and other racial/ethnic groups, NH*	−0.058	.008	−0.030	.036
	(−0.102 to −0.017)		(−0.076 to 0.016)	
_2_] *Black/African American, NH*	0.008	.482	−0.015*	.904
	(−0.014 to 0.031)		(−0.029 to −0.001)	
_3_] *Hispanic*	−0.036	.019	−0.002	.188
	(−0.066 to −0.005)		(−0.030–0.027)	
_4_] Age	−0.001*	.023	0.000	
	(−0.002 to 0)		(0.000–0.000)	
_5_] Female (male or non-binary reference)	0.123	***	0.186	***
	(0.107–0.140)		(0.167–0.205)	
[β_6_] Level change after January 2016	0.003	.760	−0.013	
	(−0.019 to 0.027)		(−0.040 to 0.015)	
[β_7_] Change in monthly trend after January 2016 (per month)	−0.019	***	−0.004	***
	(−0.026 to −0.013)		(−0.007 to −0.001)	
[β_0_] Constant	0.400	***	0.193	***
	(0.331–0.466)		(0.083–0.303)	
Observations	274 773		33 414	
R-squared	.055		.129	

Source: Source: Authors’ analysis of linked HMIS and MMIS data in NJ and PA (see text). Abbreviations: NJ, New Jersey; PA, Pennsylvania; NH, Non-Hispanic. Notes: Robust standard errors in parentheses.

*** indicates P<0.001. Due to small sample sizes, the “Asian and other racial/ethnic groups” category includes Asian, American Indian, Alaskan native, Pacific Islander, and multiple races.

**TABLE 3:** Interrupted time series analyses of the ACA Medicaid eligibility expansion on Medicaid enrollment among persons experiencing or at-risk of homelessness in NJ and PA, 2011–2016, stratified by race/ethnicity

	NJ, Black/African American NH		NJ, Hispanic		NJ, White NH		PA, Black/African American NH		PA, Hispanic		PA, White NH	
Model (Likelihood of Medicaid Enrollment)	Coefficient (95% CI)	*P*	Coefficient (95% CI)	*P*	Coefficient (95% CI)	*P*	Coefficient (95% CI)	*P*	Coefficient (95% CI)	*P*	Coefficient (95% CI)	*P*
[β_1_] Level change post-expansion	0.063 (0.021–0.107)	.006	0.044 (0.012–0.077)	.010	0.107 (0.080–0.134)	***	0.070 (0.046–0.094)	***	0.095 (0.026–0.164)	.008	0.102 (0.058–0.147)	***

[β_3_] Change in monthly trend post-expansion (per month)	0.005 (0.003–0.007)	***	0.005 (0.002–0.008)	***	0.008 (0.005–0.011)	***	0.002 (−0.003–0.007)	0.528	0.002 (−0.010–0.014)	.744	0.001 (−0.002–0.005)	.452

[β_2_] Monthly trend (per month)	−0.001 (−0.002–0.001)	.314	−0.001 (−0.002–0)	.324	−0.002 (−0.004– 0)	.025	0.006 (0.003–0.009)	0.001	0.001 (0–0.002)	.247	0.003 (0–0.007)	.051

Homelessness assistance services per year												
[β_4_] *1 month with homelessness assistance services*	0.166	***	0.133	.001	0.132	***	0.065	***	−0.061***	***	0.096	***
	(0.124–0.211)		(0.063–0.202)		(0.072–0.192)		(0.039–0.091)		(−0.079 to −0.043)		(0.057–0.136)	
[β_5_] *2+ months with homelessness assistance services*	0.191	***	0.135	.001	0.213	***	0.119	***	0.005	.845	0.157	***
	(0.129–0.254)		(0.067–0.202)		(0.157–0.268)		(0.103–0.135)		(−0.050–0.061)		(0.128–0.186)	
_4_] Age	−0.001*	.087	−0.002	.005	−0.000	.537	0.000	.295	−0.001	.218	0	.652
	(−0.003–0)		(−00.4 to −0.001)		(−0.001–0.001)		(0–0.001)		(−0.002–0.001)		(−0.001–0)	

_5_] Female male or non-binary reference)	0.125 (0.105–0.145)	***	0.157 (0.129–0.185)	***	0.097 (0.084–0.110)	***	0.195 (0.164–0.225)	***	0.194 (0.170–0.218)	***	0.168 (0.147–0.189)	***

[β_6_] Level change after January 2016	0.021 (−0.009–0.050)	.157	−0.009 (−0.036–0.018)	.472	−0.004 (−0.033–0.025)	.773	−0.016 (−0.038–0.006)	.146	−0.015 (−0.100–0.069)	.711	−0.008 (−0.051–0.034)	.691

[β_7_] Change in trend in months after January 2016	−0.020 (−0.028 to −0.012)	***	−0.018 (−0.028 to −0.008)	.001	−0.020 (−0.026 to −0.015)	***	−0.004 (−0.011–0.003)	.252	0.002 (−0.014–0.018)	.833	−0.004 (−0.01–0.002)	.194

[β_0_] Constant	0.389 (0.311–0.467)	***	0.438 (0.353–0.524)	***	0.367 (0.331–0.403)	***	0.127 (0.074–0.181)	***	0.416 (36.5–0.467)	***	0.212 (0.097–0.326)	***

Observations	136 322		50 922		78 536		13 183		2 518		16 772	
R-squared	.054		.052		.063		.148		.106		.122	

Source: Authors’ analysis of linked HMIS and MMIS data in NJ and PA (see text). Abbreviations: NJ, New Jersey; PA, Pennsylvania; HMIS, HomelessManagement Information System; MMIS, Medicaid Management Information System; NH, Non-Hispanic; Δ, “change.” Notes: Robust standard errorsin parentheses.

*** indicates P<0.001. Small sample sizes limited analysis of the “Asian and other racial/ethnic groups” category

## Data Availability

The data that support the findings of this study are available from Medicaid and homelessness services agencies in Pennsylvania and New Jersey, but restrictions apply to the availability of these data, which were used under license for the current study, and so are not publicly available. Data are, however, available from the authors upon reasonable request and with permission of Medicaid and homelessness services agencies in Pennsylvania and New Jersey.
